# Drug Distribution and Basic Pharmacology of Paclitaxel/Resveratrol-Coated Balloon Catheters

**DOI:** 10.1007/s00270-018-2018-9

**Published:** 2018-07-02

**Authors:** Ulrich Speck, Akvile Häckel, Eyk Schellenberger, Stefanie Kamann, Melanie Löchel, Yvonne P. Clever, Daniel Peters, Bruno Scheller, Sabrina Trog, Stephanie Bettink

**Affiliations:** 1 Department of Radiology, Experimental Radiology, Charité, 10098 Berlin, Germany; 2 Innora GmbH, Berlin, Germany; 3 Clinical and Experimental Interventional Cardiology, University of Saarland, 66421 Homburg, Saarland Germany; 4 Humboldt University, Berlin, Germany

**Keywords:** Drug-coated balloon catheter, Additive, Drug transfer, Efficacy, Tolerance, Preclinical

## Abstract

**Purpose:**

To experimentally investigate a new homogenously paclitaxel/resveratrol-coated balloon catheter in terms of transport of the coating to the treated tissue and local effects including histology and functional tests.

**Methods:**

Adherence of the coating to the balloon was explored by in vitro simulation of its passage to the lesion. Paclitaxel and resveratrol transfer to the vessel wall was investigated in porcine coronary and peripheral arteries. Matrix-assisted laser desorption/ionization (MALDI) was used for direct microscopic visualization of paclitaxel in arterial tissue. Inhibition of neointimal proliferation and tolerance of complete coating and resveratrol-only coating was investigated in pigs 4 weeks after treatment, and the effect of resveratrol on inflammation and healing after 3 and 7 days.

**Results:**

Drug loss on the way to the lesion was < 10% of dose, while 65 ± 13% was detected at the site of balloon inflation. After treatment similar proportions of drug were detected in coronary and peripheral arteries, i.e., 7.4 ± 4.6% of dose or 125 ± 74 ng/mg tissue. MALDI showed circumferential deposition. Inhibition of neointimal proliferation by paclitaxel/resveratrol coating was significant (*p* = 0.001) whereas resveratrol-only coating did not inhibit neointimal proliferation. During the first week after treatment of peripheral arteries with resveratrol-only balloons, we observed nominally less inflammation and fibrin deposition along with a significant macrophage reduction and more pronounced re-endothelialization. No safety issues emerged including left ventricular ejection fraction for detection of potential distal embolization after high-dose treatment of coronary arteries.

**Conclusions:**

Paclitaxel/resveratrol-coated balloons were effective and safe in animal studies. Beyond acting as excipient resveratrol may contribute to vascular healing.

## Introduction

Angioplasty has become the preferred local treatment of stenosis and occlusions in many vascular territories, offering high and immediate success, low invasiveness, and moderate cost. However, after successful initial restoration of patency, long-term benefit is hampered by high rates of restenosis—most frequently occurring 3–12 months after treatment. The main underlying mechanism is excessive neointimal proliferation in response to injury [1]. While additional stenting increases the initial lumen gain and prevents recoil, continuous irritation contributes to excessive scar formation beyond the initial healing process [2].

The efficacy of drug-eluting stents (DES) was the incentive for coating paclitaxel onto angioplasty balloons [3]. Release of paclitaxel from the balloons improves persistent patency but only at higher doses than paclitaxel released from coated stents.

Small molecules added to the coating have been shown to markedly improve the efficacy of balloon coating in both animal experiments and clinical trials [3, 4]. A variety of additives (e.g., iodinated contrast medium, urea, surfactants, plasticizers) are in use today most of them with no biological effect or even known to cause damage to cell membranes. Other factors effecting the effectiveness of paclitaxel beyond the dose and the additive are the balloon membrane and, the coating method. Some outcome data comparing the results of clinical trials investigating different drug-coated balloons have been presented at scientific meetings [5] but they are not consistent in terms of trial design, patient populations, and methodology. In the current study, we investigated a coating with resveratrol as a potentially beneficial antioxidant [6] admixed to paclitaxel. Resveratrol is an antioxidant with a variety of actions including inhibition of platelet activation and adhesion, inhibition of blood coagulation, protective effects on the endothelium, and enhancement of nitric oxide formation. Although selected because of its effects on the adherence and release of paclitaxel, resveratrol has unique additional effects that deserve further investigation.

A variety of basic features of DCB are not addressed or cannot be explored in clinical trials. The experiments reported in this paper fill a portion of this gap. In the preclinical studies presented here we investigated adherence of paclitaxel and resveratrol to the balloon on its in vitro simulated way to the lesion, drug and resveratrol transfer to coronary and peripheral vessels in pigs, inhibition of neointimal proliferation, and effects of high-dose paclitaxel/resveratrol on the vessel wall. The methods included direct microscopic visualization of paclitaxel in arterial tissue. Furthermore, the effects of resveratrol-only balloon coatings were investigated in the same animal model.

## Materials and Methods

### Experimental Strategy

Experimental models were selected according to published comparative data and investigator’s experience. Studies were performed in vitro (drug loss during passage through a hemostatic valve, sheath and blood) and in pig in coronary or peripheral arteries, clinical doses and significant overdose to detect potential desired or toxic effects not apparent at lower dose. Animals were sacrificed at three different points in time: a few minutes after treatment to measure peak drug concentrations, 3 or 7 days after treatment for early inflammatory reactions and healing and after 4 weeks for neointimal proliferation or toxicity.

Table 1 summarizes the experiments conducted, some of which were pilot studies in preparation for an initial clinical trial. Whenever possible, treatments within one experiment were performed in randomized order. To keep the number of experimental animals as low as possible, arteries in different vascular territories were treated in some pigs as indicated in the table.

**Table 1:** Survey on experiments

#	Experiment	Sampling time	Pigs	Arteries	Premounted stents on balloons	Measurements (*n*)	Results, Table nos.
1	Loss during passage through valve, sheath, and blood (in vitro)	Immediately after passage of valve, catheter, and floating in blood	0	0	No	PTA catheters = 3 PTCA catheters = 4	2
2	Drug and additive content during PTA at treatment site	During balloon inflation	*n* = 4	External iliac, *n* = 4	No	Inflated balloon + artery in situ	2
3	Drug and additive following PTA in arterial wall	Arteries: 10–30 min after balloon deflation and retraction	*n* = 4	External iliac, n = 8	No	Paclitaxel and resveratrol in the arterial wall and on balloons after inflation, deflation, and retraction	2
4	Drug and additive content following PTCA	Arteries: 10–30 min after balloon deflation and retraction	*n* = 4*	Coronary, right, left anterior descending, left circumflex; *n* = 8 arteries	No	Paclitaxel and resveratrol in the arterial wall and on balloons after inflation, deflation and retraction	2
5	MALDI Matrix-assisted laser desorption/ionization	Internal iliac arteries	*n* = 6	Internal iliac, *n* = 12 (only 1 example shown)	No	Paclitaxel displayed in histological slices	Figure 2
6	Impact of paclitaxel-resveratrol-coated balloon catheters on neointimal proliferation	4 weeks after treatment	2 treatment arms, *n* = 6/arm	External iliac/femoral, 2 treatment arms, *n* = 12/arm	Yes	Tolerance, QCA, histomorphometry	3
7	Impact of paclitaxel-resveratrol-coated balloon catheters on neointimal proliferation and ejection fraction, downstream myocardium	4 weeks after treatment	3 treatment arms, *n* = 5/arm	Coronary arteries, left anterior descending, left circumflex, 3 treatment arms, *n* = 9–10/arm; 5 hearts/arm	Yes, in high-dose group: only 1st balloon per treatment site	Effects including ejection fraction, QCA, histomorphometry of coronary arteries, histology of myocardial tissue	3, 4, 5
8	Impact of resveratrol-only-coated balloon catheters on neointimal proliferation	4 weeks after treatment	2 treatment arms, *n* = 7/arm	External iliac/femoral, 2 treatment arms, *n* = 7/arm	Yes	Tolerance, QCA, histomorphometry	3
9	Impact of resveratrol-only coating on neointimal proliferation	4 weeks after treatment	2 treatment arms, *n* = 8/arm**	Coronary arteries, right, left anterior descending, left circumflex; 11–12 arteries/arm	Yes	Tolerance, QCA, histomorphometry	3
10	Impact of resveratrol-only coating on early healing	3 or 7 days after treatment, data combined because of no recognizable difference	2 treatment arms, n = 8 (total)	External iliac/femoral, 2 treatment arms, *n* = 8/arm	No	Histology	6

*Same animals as in line 3; **including pigs of line 8

**Table 2:** Drug loss on the simulated way to the lesion and distribution of drug/additive following PTA in peripheral and coronary arteries of pigs

	*n*	Paclitaxel-resveratrol-coated balloons 5.0 or 7.0 × 40 mm, 0.035’’ guide wire lumen	Paclitaxel-resveratrol-coated balloons 3.5–20 mm, 0.014’’ guide wire lumen
Loss during passage through valve, sheath, and blood (in vitro) (% of dose)	3/4	5.1 ± 2.6	0.6 ± 6.3
Treatment site		External iliac artery	Coronary arteries
Paclitaxel at or on balloon in artery (% of dose)	4	65 ± 13	Not done
Resveratrol at or on balloon in artery (% of dose)	4	60 ± 15	Not done
Paclitaxel in arterial wall (µg) (µg/g ~ ng/mg tissue)	8	266 ± 229 145 ± 50	65 ± 28 126 ± 64
Paclitaxel in arterial wall (% of dose)	8	7.1 ± 6.1	7.8 ± 3.4
Resveratrol in arterial wall (µg)	8	22 ± 25	< LOQ
Residual paclitaxel on balloons (% of dose)	8	9.6 ± 8.7	25.4 ± 16.0
Residual resveratrol on balloons (% of dose)	8	13.8 ± 4.7	23.3 ± 12.1

*n* number of balloon catheters or arteries

### Angioplasty Balloon Catheters

**Fig. 1:**
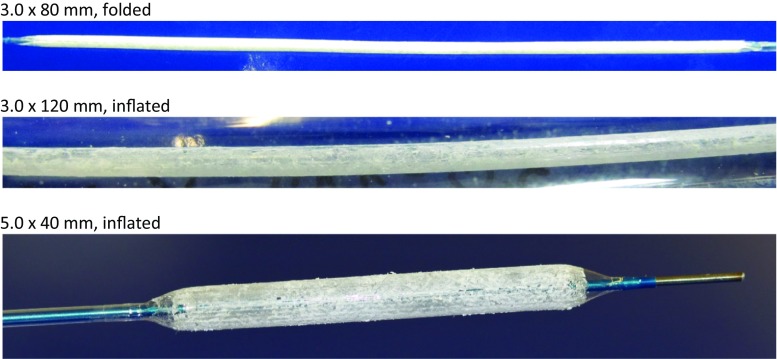
Examples of paclitaxel-resveratrol-coated balloons, 3 µg paclitaxel/mm^2^ balloon surface

The following catheters were used in the experiments: over-the-wire(OTW) design, 0.014’’ or 0.035’’ wire lumen, various balloon sizes as indicated in the Results section, either uncoated or coated in an expanded state with paclitaxel at doses of 3 or 5 µg/mm^2^ and resveratrol as an additive (Fig. 1) or coated with resveratrol only, 6.1 ± 0.2 µg/mm^2^. Inhibition of neointimal proliferation was studied using bare metal stents mounted on uncoated or coated balloons to enhance proliferation. In the high-dose coronary group (5 µg/mm^2^) a second coated balloon without premounted stent was inflated in the stented vessel segment. Vessel overstretch was approximately 10–30% of the vessel diameter.

### Adherence of Paclitaxel and Resveratrol to the Balloon on its in Vitro Simulated Way to the Lesion

Small balloons (3.5–20 mm) were passed through a hemostatic valve (on/off closure) into a blood-filled guiding catheter (Medtronic Launcher JL 3.5 6F, length 95 cm). Larger balloons (size 5.0–40 mm) were passed through a blood-filled Terumo Destination 6F introducer sheath (45 cm in length) with a membrane valve. Balloons were advanced into a vial with stirred heparinized porcine blood at approximately 37 °C, kept in blood for 1 min, placed in an empty vial, inflated, and cut off the shaft. Following ethanol (> 96%) addition, the samples were sonicated to dissolve the drug and centrifuged.

### Animal Experiments

The studies were conducted in a total of 139 arteries in 65 domestic pigs weighing 24.0–30.5 kg. All applicable institutional and/or national guidelines for the care and use of animals were followed, e.g., the Commission Directive 86/609/EEC and the German Animal Protection Act, and approved by the Animal Ethics Committee (IMTR 42502-2-916, Sachsen–Anhalt, Germany).

For details of the experiments see Kelsch et al. [7]. Briefly, anticoagulants were started 2 days before treatment and continued in animals surviving for 4 weeks until sacrifice: 75 mg clopidogrel and 100 mg acetylsalicylic acid. Long-acting verapamil was started 24 h prior to treatment to reduce vascular spasm during the procedure. The pigs were sedated before general anesthesia was induced. Access was provided through an external carotid artery. Heparin (5000 IU) and 250 mg lysine acetylsalicylate were administered intra-arterially.

Vessel segments were selected in coronary or iliac/femoral arteries. The balloons were deployed as indicated and inflated for 60 s with 8–14 atm to achieve a balloon-to-artery ratio of approximately 1.2. In the coronary study, 15 pigs were treated in the left anterior descending and the left circumflex artery—5 animals each with uncoated balloons, coated balloons (3 µg paclitaxel/mm^2^), and two coated balloons (each 5 µg/mm^2^) per vessel segment, numbers of vessels per coated balloon and uncoated control are given in Table 3. When two balloons were deployed in the same vessel segment the first one had a premounted stent and the second one did not.

**Table 3:** Inhibition of neointimal proliferation and other vascular effects of paclitaxel/resveratrol-matrix or resveratrol-only-coated balloon catheters in coronary and peripheral arteries; follow-up period: 28 days

	Paclitaxel-resveratrol-coated balloons							Resveratrol only—coated or uncoated balloons					
	Coated 3 µg/mm^2^	Coated 2 × 5 µg/mm^2^	Uncoated	p*	Coated 3 µg/mm^2^	Uncoated	p versus uncoated	Resveratrol only, 6 µg/mm^2^	Uncoated	p versus uncoated	Resveratrol only, 6 µg/mm^2^	Uncoated	p versus uncoated
	Coronary arteries, LAD and LCX,				External iliac or femoral artery			Coronary arteries			External iliac or femoral artery		
*Quantitative (coronary) angiography (QCA, QA)*													
*n* (analyzed vessels)	10	10	10		12	12		12	11**		7	7	
RFD initial (mm)	2.92 ± 0.17	2.89 ± 0.23	2.76 ± 0.31	> 0.1	5.60 ± 0.60	5.56 ± 0.48	> 0.1	2.04 ± 0.25	2.23 ± 0.24	0.09	5.67 ± 0.75	6.00 ± 1.05	0.52
Balloon size (mm)	3.5 × 20				6 or 7 × 20			3.5 × 20			6 or 7 × 20		
Overstretch (−)	1.11 ± 0.07	1.14 ± 0.10	1.15 ± 0.10	> 0.1	1.16 ± 0.18	1.20 ± 0.10	> 0.1	1.35 ± 0.16	1.27 ± 0.08	0.18	1.28 ± 0.20	1.24 ± 0.13	0.67
Neointimal thickness (mm)					0.30 ± 0.34	1.60 ± 1.37	0.004				1.76 ± 0.51	1.60 ± 0.27	0.48
LLL (mm)	0.26 ± 0.22	0.17 ± 0.24	1.12 ± 0.47	0.001				1.38 ± 0.38	1.07 ± 0.27	0.04	1.56 ± 1.41	1.68 ± 0.72	0.85
Diameter stenosis (%)	− 2.8 ± 8.9	− 6.4 ± 13.4	26.5 ± 17.7	0.001				35.3 ± 15.8	22.1 ± 13.1	0.04	− 2.7 ± 18.5	4.0 ± 20.6	0.54
*Histomorphometry*													
*n* (analyzed vessels)	10	9	10		12	12		12	11**		7	7	
Vessel diameter (mm)	3.40 ± 0.09	3.51 ± 0.10	3.13 ± 0.11	0.001	6.30 ± 0.41	6.35 ± 0.78	> 0.1						
Lumen diameter (mm)	2.85 ± 0.17	2.97 ± 0.13	1.99 ± 0.43	0.001	5.52 ± 0.48	4.87 ± 0.90	0.038						
Max neoint thickness (mm)	0.42 ± 0.13	0.37 ± 0.05	0.81 ± 0.30	≤ 0.003	0.64 ± 0.18	1.19 ± 0.53	0.003						
Neointimal area (mm^2^)	2.62 ± 0.64	2.81 ± 0.62	4.22 ± 0.97	0.002	7.48 ± 1.45	12.3 ± 5.5	0.007	3.14 ± 0.89	2.89 ± 0.72	0.464	7.11 ± 4.82	7.27 ± 1.85	0.936
Diameter stenosis (%)	16.3 ± 4.5	15.5 ± 3.2	36.5 ± 12.0	0.001	12.5 ± 2.8	23,2 ± 10.0	0.002						
Area stenosis (%)	29.4 ± 6.9	29.5 ± 5.7	57.5 ± 15.9	0.001	24.4 ± 5.0	40.6 ± 15.4	0.002						
Inflammation score (−)	2.85 ± 0.31	2.93 ± 0.20	2.80 ± 0.38	0.35	2.47 ± 0.52	2.67 ± 0.32	> 0.1	0.92 ± 0.79	1.27 ± 0.79	0.292	0.65 ± 0.50	1.21 ± 0.58	0.071

All balloons with premounted stents to further stimulate neointimal proliferation except 2 × 5 µg/mm^2^ (= 2 balloons deployed in the same vessel segment, the first one with stent, the second one without stent); negative diameter stenosis indicates preserved overstretch/enlarged vessel lumen; LLL = late lumen loss (difference between lumen diameter after treatment and 4 weeks later); *p* coated versus uncoated; p* refers to each of the two dose levels, **) one RCA too narrow, not reached/treated

In the cardiac efficacy and tolerance study, left ventricular angiography was done before any intervention and at 28-day follow-up before coronary angiography. Offline measurement of global left ventricular function was performed using the Siemens LV-EF module (Axiom Artis dBC).

After final angiography and euthanasia the hearts were examined for gross pathological anomalies. Tissue samples were taken from the left ventricle close to the septum, the right ventricle close to the right coronary artery, and the apex, and examined for histological evidence of hemorrhage, thrombosis, fibrosis, necrosis, and scar formation, as well as, evidence for endocarditis and epicarditis. Tissue slices at a thickness of 2 µm were stained with hematoxylin/eosin and then microscopically examined.

To detect potential effects of resveratrol on healing, some animals treated with resveratrol-only coated balloons were sacrificed 3 or 7 days after the intervention.

For euthanasia 10 ml supersaturated potassium chloride (25%) was intravenously injected in deep anesthesia. Treated vessel segments were dissected and either shock-frozen in cryogel (Tissue Tek O.C.T.™, Weckert, Kitzingen, Germany) or preserved in 4% formalin solution for histomorphometry or weighted and kept at − 20 °C for analysis of paclitaxel and resveratrol.

Paclitaxel was visualized with a matrix-assisted laser desorption/ionization (MALDI) [8] imaging source (MALDI LTQ XL™, Thermo Fisher Scientific) attached to a Fourier transform orbi trap mass spectrometer (LTQ Orbitrap XL™ MS, Thermo Fisher Scientific) and equipped with a mini-nitrogen laser (LTB, Lasertechnik Berlin).

### Quantitative Coronary Analysis and Histomorphometry

Briefly, the CAAS II System (Pie Medical, Netherlands) was used for quantitative vessel analysis by an experienced observer blinded to treatment groups. Stented artery segments were embedded in methyl methacrylate. Final specimens were stained with hematoxylin–eosin or Masson-Goldner. Histomorphometric measurements were taken using the “LUCIA G” image program. Injury scores were assigned as described by Schwartz et al. [9], and inflammation was graded using scores presented by Kornowski et al. [10]. If required scores were adjusted for assessment of non-stented segments.

Non-stented peripheral vessel segments treated with resveratrol-only or plain balloons were harvested after 3 and 7 days and embedded in paraffin. In addition to fibrin deposition [11], inflammation (Mac-2, clone M3/38, Cedarlane, Burlington, Ontario, Canada) and re-endothelialization (CD31/PECAM-1, clone M-20-R, Santa Cruz Biotechnology, Dallas, Texas, US) were assessed. Microscopic images were analyzed using Image J software (National Institutes of Health). Macrophages were quantified by determination of Mac-2 fluorescence intensity. The proportion of lumen circumference covered by CD31-positive cells was estimated in increments of 5% to assess re-endothelialization. Values are means of two sections from three section levels each.

### Analysis of Paclitaxel and Resveratrol

#### Unused and Used Coated Balloons

Paclitaxel and resveratrol content was determined by placing unused or used balloons in a vial and adding ethanol for extraction.

#### Arteries (Treated Vessels Including Adjacent Segments, in Some Peripheral Arteries Including Inflated Balloons)

For extraction, a defined volume of ethanol was added. The samples were homogenized (Precelly 24 Dual Homogenizer, PEQLAB Biotechnologie GmbH, Erlangen, Germany).

#### Measurement of Paclitaxel and Resveratrol Content by HPLC/UV

Paclitaxel and resveratrol were determined by high-performance liquid chromatography (HPLC) with ultraviolet detection. Column: C18, 5 μm, 25 cm × 4.6 mm. Mobile phase: 45% phosphate buffer 0.005 M (pH 3.5) and 55% acetonitrile, 1 ml/min; retention time: paclitaxel ~ 12 min, resveratrol ~ 3 min.

### Statistical Analysis

Quantitative parameters were compared across treatment groups. Continuous variables were compared by ANOVA using the software package SPSS 15.0 for Windows (SPSS Inc., Chicago, IL, USA). Data are presented as mean ± SD.

## Results

Paclitaxel/resveratrol-matrix-coated angioplasty balloon catheters (Fig. 1) are used for the first 7 investigations listed in Table 1 and, in study 6 and 7, compared to the respective uncoated balloon catheters.

### Paclitaxel and Resveratrol Distribution

Paclitaxel and resveratrol distribution during and following PTA are summarized in Table 2. Advancement of the DCB through a hemostatic valve, an introducer sheath or guiding catheter and contact of the folded balloon with flowing blood were simulated in vitro; the loss of drug was 5.1 ± 2.6% of dose for 5.0 or 7.0 mm balloon diameter and 0.6 ± 6.3% for 3.5 mm diameter.

Next, total inflated balloons along with the adjacent arterial wall were dissected and analyzed. At this point, 65 ± 13% of paclitaxel and 60 ± 15% of resveratrol were found at the target site trapped between the balloon membrane and the endothelial surface of the arterial wall. The difference (35%) appears to have been lost before or during balloon inflation before the blood flow is interrupted.

Following balloon deflation and retraction, residual paclitaxel on the balloons was 9.6 ± 8.7% (peripheral) and 25.4 ± 16.0% (coronary). Similar proportions were found for resveratrol. Balloons in the external iliac (or femoral) and coronary arteries transferred similar proportions of paclitaxel into the arterial wall, 7.1 ± 6.1 and 7.8 ± 3.4% of dose, resulting in concentrations of 145 ± 50 and 126 ± 64 ng/mg tissue. Contrary to the findings for paclitaxel, no or only small amounts of resveratrol were detected in tissue. In summary 5% of paclitaxel was lost at the valve and in the sheath/blood before inflation and, 30% during inflation (unfolding of the balloon before blood flow is blocked), 65% was still found after inflation of the balloon, 48% of dose is washed away during deflation and retraction of the balloon and 10% remained on used balloons. The largest proportion of the drug is lost during deflation and retraction of the balloon (48%) or is never released (10%).

**Fig. 2:**
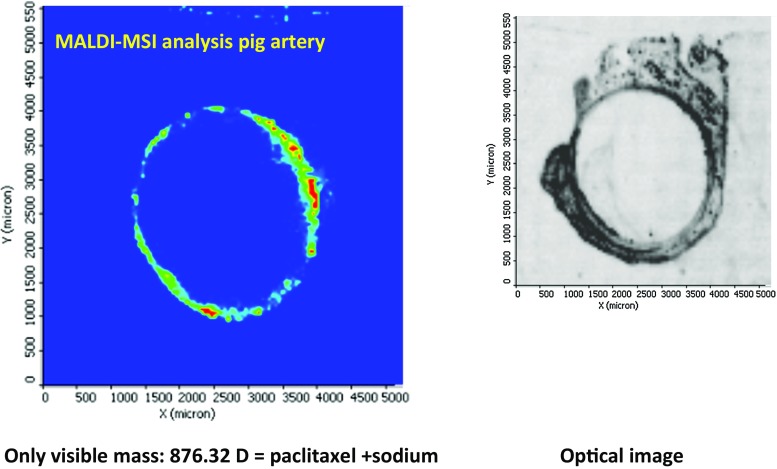
Paclitaxel in peripheral artery of a pig approx. 15 min after expansion and removal of a paclitaxel 3 µg/mm^2^/resveratrol–coated balloon. Only visible mass: 876.32 *D* = paclitaxel + sodium Optical image

Paclitaxel was identified in histological slices of arteries prepared from deep-frozen samples by matrix-assisted laser desorption/ionization. Figure 2 shows the circumferential distribution in a peripheral artery shortly after treatment with a 5.0 × 40 mm balloon.

### Inhibition of Neointimal Proliferation

Two studies investigated on paclitaxel/resveratrol-matrix-coated versus uncoated balloon catheters—one in coronary and one in peripheral arteries (Tables 1 and 2). Quantitative coronary angiography indicated minimal narrowing of the paclitaxel-treated vessel segments (late lumen loss 0.26 ± 0.22 mm) at the regular dose level and almost the same (0.17 ± 24 mm) at the high dose compared to late lumen loss of 1.12 ± 0.47 mm for use of uncoated balloons (*p* = 0.001). This finding was confirmed by histomorphometry, showing neointimal thickness of 0.42 ± 0.13 mm, 0.37 ± 0.05 mm, and 0.81 ± 0.30 mm, respectively (each of two dose levels vs uncoated: *p* ≤ 0.003).

Two peripheral arteries each were treated in 13 animals; one animal treated with uncoated balloons died from internal bleeding caused by perforation of the left iliac artery. In the peripheral arteries, the larger stents were easily visible. Therefore, the layer between the contrast-enhanced lumen and the stent was assessed by quantitative angiography and reported as ‘neointimal thickness’ in Table 2. The difference between coated (0.30 ± 0.34 mm) and uncoated balloons (1.60 ± 1.37 mm, *p* = 0.004) indicates suppression of neointimal proliferation by balloon coating.

### Local Effects of Resveratrol

In a similar experiment as described in the last paragraph, 8 animals were treated with high-dose resveratrol-only-coated balloons (Table 2). Four weeks after balloon dilatation and stent implantation, results were the same as for coated and uncoated balloons in terms of late lumen loss of coronary (1.38 ± 0.38 vs. 1.07 ± 0.27 mm) or peripheral arteries (1.56 ± 1.41 vs. 1.68 ± 0.58 mm). Histomorphometry did not reveal differences to vessels treated with uncoated balloons either. Compared to treatment with uncoated balloons, the inflammation score seemed to be somewhat reduced in both vessel territories, but the difference was not statistically significant.

**Fig. 3:**
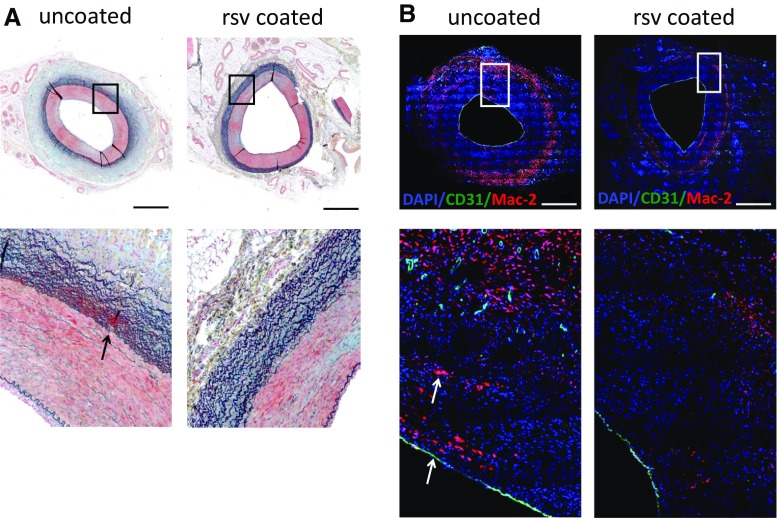
Representative histological sections of balloon-treated peripheral arteries 7 days after intervention **A** Movat pentachrome staining. Blue—ground substance; red—muscle; bright red—fibrin; black—elastic fibers. Arrow indicates fibrin deposition. **B** double immunofluorescence staining. Blue—nuclei; green—endothelial cells (CD31); red—macrophages (Mac-2). Arrows indicate luminal endothelial monolayer and macrophages. Ruler ≈ 1 mm

**Fig. 4:**
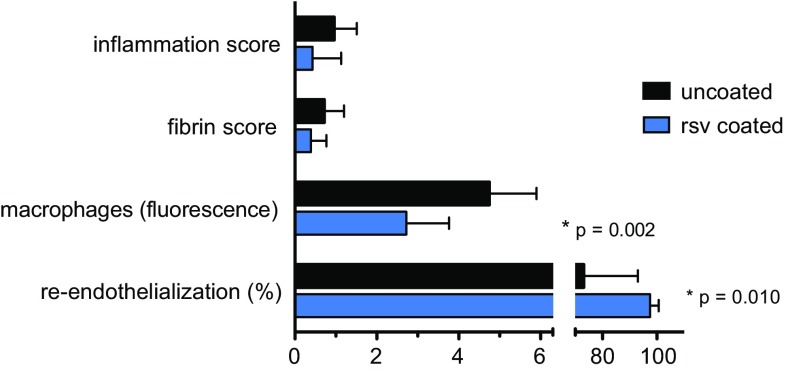
Histological analysis of 8 peripheral arteries up to 1 week after treatment with uncoated or resveratrol-only-coated balloon catheters

To further elucidate the role of resveratrol, vessels of an additional 2 animals each were harvested 3 days and 7 days after treatment with uncoated or resveratrol-only-coated balloons. Since histological parameters were consistent at different time points investigated these data were combined for comparison of treatments (Table 3). Compared to arteries treated with uncoated balloons, histology (Figs. 3, 4) of resveratrol-treated arteries revealed a tendency toward reduced fibrin deposition and significantly reduced number of macrophages (*p* = 0.002) as well as enhanced re-endothelialization (*p* = 0.01).

### Tolerance

Overall, 24 animals were treated with coronary and/or peripheral paclitaxel/resveratrol-matrix-coated balloon catheters, 5 of them with high-dose coronary balloons; in the latter group 2 vessels each were treated with two balloons in the same vessel segments. No thrombi, thrombotic occlusions, or outflow obstructions were observed. None of the DCB-treated animals died, ECG changes were of no clinical significance, only blood pressure dropped somewhat during anesthesia and following nitroglycerin injection.

In the study including the high-dose coronary group, left ventricular ejection fraction was measured before treatment and after 4 weeks. It dropped in the control group (uncoated balloons) by 6.4 ± 4.2%, and in the regular and high-dose groups by 2.5 ± 8.1 and 5.4 ± 7.0%, respectively (Table 4). Histopathology of the hearts treated with 2 high-dose balloons in two coronary arteries each revealed some increase in very slight to moderate microscopic lesions compared to the groups treated with one uncoated or one normal-dose balloon/vessel (Table 5).

**Table 4:** Left ventricular ejection fraction (%) of 5 pigs per group before treatment and at 28-day follow-up after treatment of two coronary arteries with paclitaxel/resveratrol-coated balloons (3.5 × 20 mm)

Groups	Coated 3 µg/mm^2^	Coated 2 × 5 µg/mm^2^	Uncoated	*p*
EF before treatment (%)	42.0 ± 8.3	40.1 ± 4.4	45.4 ± 9.9	0.573
EF at 28 days (%)	39.5 ± 6.5	34.7 ± 4.3	39.0 ± 6.2	0.375
Delta EF (%)	− 2.5 ± 8.1	− 5.4 ± 7.0	− 6.4 ± 4.2	0.642

Balloons with premounted stents except 2 × 5 µg/mm^2^ (= 2 balloons deployed in the same vessel segment, the first one with stent, the second one without stent). Delta EF is the difference from day 28 to before treatment

**Table 5:** Histopathological findings in myocardial tissue after treatment of two coronary arteries with up to 4 high-dose paclitaxel/resveratrol-coated 3.5 × 20 mm balloons

Treatment/number of hearts	Region	Edema	Inflammation	Vasculitis	Thrombosis	Scar
2 balloons, 3 µg Ptx/mm^2^ each *n* = 5	LV	1 × v.slight 3 × slight	1 × v.slight 1 × slight			
	Apex	3 × v.slight 2 × slight	2 × slight	1 × slight		
	RV	3 × v.slight 1 × slight	1 × v.slight			
4 balloons, 5 µg Ptx/mm^2^ each *n* = 5	LV	3 × slight		3 × v.slight 1 × slight	1 × slight	1 × v.slight 2 × slight 1 × moderate
	Apex	5 × slight	1 × v.slight 1 × slight	1 × v.slight 1 × slight		1 × moderate 1 × slight
	RV	1 × v.slight 1 × slight				
2 balloons uncoated *n* = 5	LV	3 × slight	1 × slight			
	Apex	3 × slight 1 × moderate	1 × v.slight			
	RV	1 × v.slight 2 × slight				

Same animals as in Table 3; *LV* left ventricle, *RV* right ventricle (not treated), *Ptx* paclitaxel, *v. slight* very slight

**Table 6:** Histology of peripheral arteries 3 or 7 days (data pooled) after treatment with resveratrol-only-coated balloon catheters (6 or 7 × 60 mm)

Analysis parameter	Balloon area		*p*
	Uncoated	Resveratrol-coated	
*n* (vessels)	8	8	
Injury score	0.29 ± 0.47	0.01 ± 0.03	0.131
Intima + media (mm^2^)	4.00 ± 1.13	3.62 ± 0.73	0.440
Inflammation score	0.96 ± 0.55	0.43 ± 0.70	0.118
Fibrin score	0.72 ± 0.48	0.39 ± 0.38	0.145
Macrophages (fluorescence)	4.75 ± 1.15	2.72 ± 1.04	0.002
Re-endothelialization (%)	73.1 ± 19.9	97.5 ± 3.2	0.010

No stents, *n* value = number of arteries included; macrophages: relative fluorescence of immunofluorescent stained Mac-2; values are mean ± SD; *p* value determined by Student’s *t* test

Sixteen DCB-treated animals were observed for 4 weeks. All animals gained weight and showed no signs of weakness or disease. Reangiography revealed no vascular abnormalities such as thrombotic occlusions or aneurisms in the paclitaxel-treated animals.

## Discussion

### Local Drug Delivery

A variety of studies was performed to explore relevant properties of a clinically available DCB with the additive resveratrol known for beneficial vascular and cardiovascular effects.

Inhibition of injury-induced neointimal proliferation by drugs has been shown in randomized clinical trials for oral administration [12, 13] and various ways of local drug delivery including drug-eluting stents, drug-coated balloons, and liquid preparations [14]. Effective therapeutic agents are paclitaxel and limus drugs such as sirolimus or everolimus. The latter are preferred on coronary stents. However, although certain dose ranges have been established for different devices and procedures, little is known about the required drug concentration in tissue and how it can be achieved. Shortly after balloon deployment, we measured mean paclitaxel concentrations of 145 µg/g in porcine iliac and 126 µg/g in coronary arteries, both distinctly above effective drug concentrations [15, 16]. Far less resveratrol is transferred to the arterial wall and—considering its lower biological effect on cell proliferation—it is not surprising that no antirestenotic effect is observed with resveratrol coating without paclitaxel.

### Dose Considerations

Effective paclitaxel doses differ for different modes of administration, e.g., (a) for coronary stents in the range of 100–200 µg (Taxus™, only 10% released), (b) ca. 110 µg if dissolved paclitaxel is infused into short separated segments of coronary arteries [14], and to (c) 500–1000 µg on drug-coated coronary balloons. Data on tissue concentrations following DES placement are scarce but concentrations seem to be < 1 µg/g [17]. It is unknown how much of the drug from a DES stays in the tissue and for how long and which proportion is rapidly cleared by the blood stream.

The need for a higher dose on drug-coated balloons compared to stents may be explained by the limited transfer of the drug from the balloon to the vessel wall. Our results with the resveratrol-matrix-coated DCB indicate that the largest proportion of the drug reaches the target site and is pushed against the vessel wall. Because solubility is very low, diffusion of the drug into tissues has no role. Drug particles are pushed against and into the tissue. A large proportion is washed away during deflation and retraction of the balloon. The tissue concentration following DCB deployment by far surpasses the solubility of the drug in aqueous media. Water-solubility of paclitaxel is 0.30 ± 0.02 µg/ml, corresponding to 0.3 µg/1 g water [19]. We found a concentration of 145 ± 50 µg/g in coronary and 126 ± 64 µg/g in peripheral arteries (Table 3), far exceeding the solubility in tissue water.

This concentration is due to solid drug reservoirs, which are the source of long-lasting low tissue concentrations and efficacy, whereas the non-dissolved solid drug is inefficacious and nontoxic.

Although capillary embolism was detected on a microscopic level no relevant downstream morphological or physiological effects were seen in the animal studies in spite of high dose which is in full agreement, e.g., with the lack of troponin increase in DCB-treated patients suffering from in-stent restenosis [18].

### Resveratrol

Another subject of the current investigation is resveratrol, the additive to paclitaxel. Previous comprehensive experiments and published data [20–24] suggest resveratrol to be a useful coating additive. The primary purpose of the additive is to modulate adherence and release of the drug. This is fulfilled by resveratrol, since drug loss during passage of the hemostatic valve and sheath and during floating in blood was < 10% of dose compared to > 20% of the currently leading products in similar experiments [7] and residual paclitaxel on the balloon after PTA was approximately 10%. Diminished drug loss may be explained by the lower water-solubility of resveratrol as compared to X-ray contrast agent or urea (additives to the above mentioned DCB [7]). Antioxidants in general and resveratrol in particular are known for their beneficial biological effects [24], especially for protecting endothelium [25–29]. Consistent with these reports are our findings on reduced inflammation, fibrin deposition, presence of macrophages, and accelerated re-endothelialization. However, neither the relevance of the pharmacological effects of resveratrol to paclitaxel-coated balloons nor the impact on clinical findings has yet been explored.

A direct effect on neointimal proliferation, as observed in carotid arteries of rats after implantation of resveratrol-coated stents [27] or after exposure of femoral arteries of rabbits to resveratrol solutions [23], was not seen in porcine arteries treated with resveratrol-only-coated balloons.

### Limitations

The majority of the studies was planned and performed in preparation of a first clinical trial on paclitaxel/resveratrol-coated balloon catheters. They address the safe transfer of the drug to the treatment site, tolerance and efficacy of the coating in a large animal model but they did not address detailed pharmacokinetics of paclitaxel and resveratrol nor the mechanism of action. Emphasis is on information which cannot be obtained in clinical trials, such as local pharmacokinetics and histology. As usual in preclinical studies number of large animals was limited and in this study the animals were young and healthy. Results may not reflect the findings in patients and are no substitute for randomized clinical trials in a large patient population.

## Conclusions

According to the findings of in vitro and animal experiments paclitaxel/resveratrol-coated DCB carry the drug to the lesion and, during inflation, transfer a sufficient portion of the dose to the adjacent vessel wall to inhibit neointimal proliferation. A larger proportion is washed away during deflation and retraction. The coating is well tolerated. Reveratrol may have a beneficial effect on inflammation and reendothelization.
